# Perceived Enablers and Barriers to Optimal Health among Music Students: A Qualitative Study in the Music Conservatoire Setting

**DOI:** 10.3389/fpsyg.2017.00968

**Published:** 2017-06-28

**Authors:** Rosie Perkins, Helen Reid, Liliana S. Araújo, Terry Clark, Aaron Williamon

**Affiliations:** ^1^Centre for Performance Science, Royal College of MusicLondon, United Kingdom; ^2^Faculty of Medicine, Imperial College LondonLondon, United Kingdom; ^3^Guildhall School of Music and DramaLondon, United Kingdom

**Keywords:** conservatoire, health promotion, higher education, music, musicians, wellbeing

## Abstract

Student health and wellbeing within higher education has been documented as poor in relation to the general population. This is a particular problem among students at music conservatoires, who are studying within a unique educational context that is known to generate both physical and psychological challenges. This article examines how conservatoire students experience health and wellbeing within their institutional context, using a framework from health promotion to focus attention on perceived enablers and barriers to optimal health in relation to three levels: lifestyle, support services, and conservatoire environment. In order to respond to the individuality of students’ experiences, a qualitative approach was taken based on semi-structured interviews with 20 current or recent conservatoire students in the United Kingdom. Thematic analysis revealed a complex set of enablers and barriers: (i) *lifestyle enablers* included value placed on the importance of optimal health and wellbeing for musicians and daily practices to enable this; *lifestyle barriers* included struggling to maintain healthy lifestyles within the context of musical practice and learning; (ii) *support enablers* included accessible support sources within and beyond the conservatoire; *support barriers* included a perceived lack of availability or awareness of appropriate support; (iii) *environmental enablers* included positive and enjoyable experiences of performance as well as strong relationships and communities; *environmental barriers* included experiences of comparison and competition, pressure and stress, challenges with negative performance feedback, psychological distress, and perceived overwork. The findings reveal a need for health promotion to focus not only on individuals but also on the daily practices and routines of conservatoires. Additionally, they suggest that continued work is required to embed health and wellbeing support as an integral component of conservatoire education, raising awareness so that all students are fully informed of where, and how, to seek the information or help that they may need. Finally, they indicate a need for more radical scrutiny of the cultures of conservatoires and an assessment of how these can be modified to best optimize students’ health and wellbeing.

## Introduction

Optimizing health and wellbeing has been a focus within higher education for many decades (Warner, 1984). Around the turn of the 21st century, research indicated that the health of students in higher education – and particularly their emotional health – was poor in relation to age-matched peers (Stewart-Brown et al., 2000). More recently, Storrie et al. (2010) corroborate that student health tends to be poor in relation to the general population, with emotional health a greater problem than physical health. Indeed, psychological distress rates are reported as high among higher education students from around the world (Bewick et al., 2010; Stallman, 2010; Ibrahim et al., 2013). This article focuses specifically on the context of students studying at United Kingdom music conservatoires, responding to Storrie et al.’s (2010) point that student concerns around health tend to be institution-specific.

In the United Kingdom, conservatoires are tertiary institutions that specialize in educating performing artists, including musicians. Music students at conservatoires typically specialize in one principal musical activity (such as performance, composition, or conducting) and receive a large proportion of their education via one-to-one lessons with a specialist teacher. As such, conservatoires are unique educational contexts, and the health and wellbeing of conservatoire students has received growing attention. Kreutz et al. (2008) reported on musculoskeletal and non-musculoskeletal problems amongst 273 music students drawn from two United Kingdom conservatoires. Their analysis revealed that over 10% of students reported above average or severe sleep disturbance, inappropriate tiredness, weather sensitivity, concentration problems, or headaches requiring medication. Additionally, 48% of the sample reported above average to severe musculoskeletal pain in at least one bodily site. Indeed, that musculoskeletal pain is a frequent experience for conservatoire music students is supported by a number of other studies (Spahn et al., 2002; Hagberg et al., 2005; Ackermann et al., 2011; Steinmetz et al., 2012). Furthermore, the mental health of higher education music students has also received scrutiny. Hildebrandt et al. (2012), for example, reported that fatigue, depression, and stage fright increased significantly over the 1st year of higher education in three Swiss music hochschule, while Kaspersen and Gotestam (2002) found high levels of music performance anxiety (MPA) among Norwegian conservatoire students, particularly for pianists and string players. Similarly, Papageorgi et al. (2013) found that musical performance anxiety was a concern for the majority of the 244 United Kingdom-based undergraduate student and professional musicians they sampled and that musical genre differentially impacted upon musicians’ experiences of performance anxiety, with those identifying as Western classical musicians reporting the highest levels of performance anxiety.

What, then, is it about studying music at a conservatoire that can lead to physical and psychological stress? In addition to risk factors associated with studying in higher education – such as being a full-time student, being under financial strain, and being a young person (Stallman, 2010) – Demirbatir (2015) argues that the environment of studying music “can be considered as a stressful place because of high competition, isolation, failure to achieve career goals, authoritarian teaching style, and intolerance against errors caused by stress or anxiety and financial uncertainty.” Indeed, a number of studies have attempted to scrutinize the cultures, or learning environments, of music in higher education. Nettl (1995) highlighted the emphasis placed on the performing (rather than academic) aspect of music, a point reinforced by Kingsbury’s (2001) analysis that “conservatoire life is about talent” (p. 59), contributing to hierarchies constructed through and by performing success. Perkins (2013a,b) supported Kingsbury’s analysis, reporting evidence of musical hierarchies and vocational position taking within a case study of a United Kingdom conservatoire. Furthermore, Davies (2004) reinforced the idea that learning opportunities are not always evenly distributed for conservatoire students, while Juuti and Littleton (2010) argue that the conservatoire is inherently a competitive space. Despite this context and the large number of studies identifying ill health in conservatoire music students, there has been little attempt to explore the links between the daily practices and environments of conservatoire students and aspects of their health and wellbeing.

To begin to address this gap, we first need to identify a conceptual starting point that facilitates exploration of the link between health and wellbeing, on the one hand, and the environments of conservatoires on the other. We can be guided by the large literature on health promotion, specifically within higher education contexts (see Chesky et al., 2006). Here, health promotion has been steered by a *healthy settings* approach. This means looking at the ‘whole system’ of a setting – such as a conservatoire – in order to ‘integrate a commitment to health into the fabric of settings – within their cultures, structures, processes and routine life’ (Doherty and Dooris, 2006, p. 42). This approach has underpinned initiatives such as the United Kingdom’s Healthy Universities Network, established in 2006 to offer a ‘facilitative environment for the development of a whole university approach to health and wellbeing’^1^. Indeed, in 2015, the Healthy Conservatoires Network was launched^2^.

Linked with this healthy settings approach is the concept of health literacy. As defined by Sørensen et al. (2012), health literacy is ‘concerned with the capacities of people to meet the complex demands of health in a modern society’ (p. 1). Nutbeam (2000) argues that health literacy can be thought of as an outcome of health promotion, referring to individuals’ health-related knowledge, attitudes, behavioral intentions, and personal skills. Alongside these, he also lists other outcomes that take into account the context beyond the individual, including social action and influence (social norms, public opinion) and health public policy (resource allocation, organizational practices). Essential here is that health promotion does not only involve individuals, but is a complex interaction of individuals with communities with organizations. Nutbeam (2000) goes on to suggest that there are three ways in which health outcomes can be modified: healthy lifestyles, effective health services, and healthy environments.

Within research on conservatoire health promotion to date, several studies have focused on individual behaviors and lifestyle indicators. Kreutz et al. (2009), for example, reported on the health promoting behaviors of music students at two United Kingdom conservatoires. Their analysis demonstrated that the students in their sample focused more strongly on psychosocial than physical aspects of their health, particularly neglecting their health responsibility. Further, Ginsborg et al. (2009) compared music performance students with non-music performance students in higher education, demonstrating that the music students rated their health worse than the non-music students and also scored lower on behaviors of health responsibility, physical activity, and spiritual growth. Rickert et al. (2015) also found that there is poor health awareness and knowledge of injury among student cellists. Nonetheless, of almost 200 music students surveyed in Germany, half had actively sought professional help due to playing-related health problems (Spahn et al., 2002), and music students have reported more conviction than students in other disciplines that they can influence their own health (Spahn et al., 2004).

A further body of literature has focused on health promotion programs within conservatoires (Chesky et al., 2006). A wide range of programs have been documented (see for example Williamon and Thompson, 2006; Manchester, 2007) and many conservatoires now offer core provision in aspects of playing-related health and wellbeing covering topics such as hearing health, healthy practice habits, diet and exercise, and mental wellbeing. While evaluations of such programs or other interventions remain limited, there is some evidence that preventative programs may influence psychological, although not physical, health (Zander et al., 2010). Despite this, there is acknowledgment that “it is difficult, however, to avoid risk factors beyond the individual musician’s control, such as inadequate conditions in the workplace and the psychosocial stress that may arise from working for long hours in close proximity to colleagues” (Ginsborg et al., 2012, p. 357). There is a need, then, for research that attempts to understand health promotion in the specific setting of the conservatoire in relation not only to what Nutbeam (2000) would refer to as healthy lifestyles and health or support services, but also in relation to the environments of these distinct institutions.

This article examines how conservatoire students in the United Kingdom experience health and wellbeing within the context of their institution. Specifically, it explores perceived enablers and barriers to optimal health and wellbeing as they are described and experienced by students. In so doing, it attempts to scrutinize not only individual behaviors and lifestyles, but also how these behaviors intersect with, and are constructed by, students’ experiences of health services and their perceptions of the environment within their institution. Taking a health promotion approach, we note that the field of musicians’ health has tended to use a diagnostic and clinical lens to focus predominantly on negative characteristics of musicians’ health, rather than focusing on musicians’ strengths (Ascenso et al., 2017). As a result, we place emphasis not only on barriers to optimal health and wellbeing but also on enablers, and the study was guided by the following research question: How do music students in the United Kingdom perceive the enablers and barriers that they experience in relation to their health and wellbeing while studying at a conservatoire? In addressing this question, we aim to fill a gap in existing knowledge, namely that research into musicians’ health and wellbeing has not yet focused sufficiently on students’ perceptions of enablers and barriers to their optimal health within the conservatoire setting. Moreover, our framework from health promotion places unique emphasis not only on aspects of lifestyle and support services but also on the environments in which conservatoire students study and perform.

## Materials and Methods

This study forms part of Musical Impact (2013–2017), an interdisciplinary project investigating the health and wellbeing of musicians studying and working in Europe. The project has three core strands: (1) Fit to Perform explores the attitudes, perceptions, and behaviors of musicians toward health and wellbeing, as well as their experience of chronic and acute health problems and their general fitness for performance; (2) Making Music investigates the physical and mental demands faced by musicians as they practice and perform; and (3) Better Practice examines strategies for promoting health effectively in music educational and professional contexts. This article arises from Fit to Perform, and specifically, focuses on the enablers and barriers of optimal health and wellbeing identified by conservatoire students.

### Participants

Twenty musicians studying or recently graduated from six United Kingdom conservatoires participated in the study (*n* = 15 women; *n* = 5 men), as detailed in **Table 1**. Participants were recruited via email and social media advertisements circulated by the research team, inviting conservatoire students to take part in a study investigating musicians’ attitudes toward, and experiences of, health and wellbeing. Participants self-selected to take part. Ethical approval was granted by the Conservatoires UK Research Ethics Committee (CUK REC), and participants provided written informed consent. No payment was given in exchange for participation.

**Table 1:** Summary of participant characteristics.

ID	Year of study	Principal instrument	Genre	Age range
S1001	UG3	Flute	Classical	20–24
S1003	UG3	Guitar	Jazz	20–24
S1004	UG3	Clarinet	Classical	20–24
S1005	UG3	Cello	Classical	20–24
S1006	PG2	Voice	Classical	25–30
S1007	Doctoral student	Performance research	N/A	25–30
S1008	UG3	Trombone	Classical	20–24
S1009	UG2	Viola	Classical	<20
S1010	PG3	Conducting	Classical	Not specified
S1011	UG4	Cello	Classical	25–30
S1013	PG2	Voice	Classical	20–24
S1014	Doctoral student	Performance research	N/A	35–40
S1015	UG1	Trumpet	Classical	20–24
S1016	Graduate	Clarinet	Classical	20–24
S1017	UG2	Violin	Classical	20–24
S1018	UG1	Saxophone	Classical	<20
S1019	UG1	Guitar	Classical	<20
S1021	UG2	Clarinet	Classical	20–24
S1022	UG2	Voice	Classical	20–24
S1023	UG3	Flute	Classical	20–24


*UG, undergraduate; PG, postgraduate; N/A, not applicable.*

### Procedure and Methods

The study was qualitative in design, rooted in social constructionism. This meant that we acknowledged that students’ experiences of health and wellbeing, and their attitudes toward it, would be idiosyncratic. We sought to understand how students described their own health and wellbeing, and how they situated and contextualized this within their life at the conservatoire. This permitted an inductive form of knowledge creation, in which students’ own perceptions were allowed to emerge without a-priori assumptions as to what would be identified as an enabler or a barrier at lifestyle, support or environmental levels. The knowledge generated was, therefore, assumed to be constructed in the interaction between participant and researcher, and necessarily specific to this particular sample of students. While generalizability is not possible, the study provides an in-depth and participant-driven account of students’ perceptions and, therefore, provides the basis for ongoing studies, including quantitative surveys.

Data were collected through semi-structured interviews, conducted individually at a time and location convenient to each participant. The interview schedule incorporated four main areas: (i) background and contextual information, including musical background and daily life at the conservatoire, (ii) individual attitudes toward health and wellbeing in conservatoires, (iii) perceived enablers of health and wellbeing, and (iv) perceived barriers to health and wellbeing. The interview schedule is presented in the Supplementary Material and was administered flexibly to allow the interviewer to respond to and follow-up points of interest raised by the participant. Interviews were conducted by a member of the research team and lasted between 15 and 47 min. All were face-to-face interviews, with the exception of one which was conducted by videophone. Interviews were audio recorded with permission and fully transcribed.

### Analysis

Transcripts were analyzed using a thematic inductive approach, which proceeded in four steps. First, transcripts were read for accuracy to the recording and for familiarity. Second, data were coded using NVivo10, with chunks of text that conveyed relevant meaning in relation to the research question detected by the first author and given an identifying label. Third, codes were reviewed across participants to ensure consistency, before being clustered to form sub-themes. Sub-themes were only classed as such when they were represented across at least half of the participants. Finally, sub-themes were clustered once more in order to arrive at overarching themes that characterize the enablers and barriers identified by the students. Codes and sub-themes emerged inductively in a bottom-up fashion from the data. In order to narrow the focus of enquiry, the final overarching themes were guided by a focus on enablers and barriers linked to lifestyle (daily practices and individual behaviors), health services (perceptions and experiences of support services for musicians’ health and wellbeing), and the conservatoire environment (perceptions and experiences of the conservatoire environment as it relates to health and wellbeing) (Nutbeam, 2000).

Further steps were taken to ensure the reliability of the analysis and resulting themes and sub-themes. The coding of the first interview transcript was subjected to detailed discussion by the entire research team. A second team discussion reviewed the emerging themes midway through the analysis process. Finally, a second member of the research team cross-checked all of the final themes and sub-themes for accuracy. In the case of disagreement at any of these points, consolidation was sought through discussion and cross-referencing with the raw data.

## Results

Eighteen sub-themes emerged from the analysis, clustered into lifestyle enablers and barriers (**Table 2**), support service enablers and barriers (**Table 3**), and environmental enablers and barriers (**Table 4**). In what follows, each of these themes is presented via its constituent sub-themes, with evidence provided in the form of indicative quotations and with reference to the number and percentage of participants that contributed to each sub-theme. Interpretation of the findings is reserved for the following discussion.

**Table 2:** Lifestyle enablers and barriers.

Sub-theme	Description based on codes
**ENABLERS**	
(1.1) Awareness of health	Enablers associated with perceived importance of health and wellbeing for musicians and of knowing one’s own body
(1.2) Healthy lifestyle choices	Enablers associated with healthy lifestyle choices, including exercise, healthy eating, and adequate sleep
(1.3) Helpful practice and learning strategies	Enablers associated with musicians’ daily practice and learning, including aiming for a broad portfolio of activities, the support of instrumental teachers, and practice strategies
(1.4) Promoting psychological wellbeing	Enablers associated with strategies for looking after psychological wellbeing, including individual coping strategies
**BARRIERS**	
(1.5) Lifestyle challenges	Barriers associated with daily lifestyle as conservatoire music students, including unhealthy eating habits, exercise, alcohol intake, irregular schedules, and financial strain
(1.6) Physical playing- related problems	Barriers associated with playing-related physical problems or body misuse
(1.7) Practice and learning challenges	Barriers associated specifically with musicians’ daily practice and learning, including isolation or loneliness and challenges with student-teacher relationships


*Sub-themes are presented in order of qualitative strength.*

**Table 3:** Support services enablers and barriers.

Sub-theme	Description based on codes
**ENABLERS**	
(2.1) Support sources	Enablers associated with identification and use of support sources, such as Alexander Technique, instrumental teachers, medical professionals, or conservatoire welfare staff
(2.2) Conservatoire-wide provision	Enablers associated with perceived care and support for health and wellbeing within the conservatoire, including specific initiatives
**BARRIERS**	
(2.3) Lack of sufficient support	Barriers associated with a perceived lack of, or lack of awareness of, health and wellbeing support
(2.4) Low levels of health awareness	Barriers associated with low levels of health awareness


*Sub-themes are presented in order of qualitative strength.*

**Table 4:** Environmental enablers and barriers.

Sub-theme	Description based on codes
**ENABLERS**	
(3.1) Performance success and enjoyment	Enablers associated with performing as a source of enjoyment and achievement
(3.2) Relationships and networks	Enablers associated with strong networks and supportive communities
	
**BARRIERS**	
(3.3) Comparison and competition	Barriers associated with perceived comparison to others and/or perceived competition, with negative emotional consequences
(3.4) Pressure and stress	Barriers associated with conservatoire life being perceived as high pressured and/or stressful
(3.5) Psychological distress	Barriers associated with psychological distress or mental illness
(3.6) Challenges with performance feedback	Barriers associated with the immediacy and/or negativity of performance feedback, leading to a loss of confidence
(3.7) Workload	Barriers associated with perceived overwork, particularly in relation to practice


*Sub-themes are presented in order of qualitative strength.*

### Lifestyle Enablers and Barriers

#### Lifestyle Enablers

Almost all of the participants reported an *awareness of health and wellbeing* in their lifestyles (sub-theme 1.1, *n* = 19, 95%). Of these, 18 (90%) perceived that health and wellbeing is important to them as musicians:

You can’t place the importance on [health and wellbeing] enough really. If you’re not fit and healthy you can’t perform to the best of your ability, and if you’re not a sort of happy human then you’re not going to be a happy musician. I think you’ve got to place your health and wellbeing first really, although it can actually quite often be when you’re absolutely crazy busy with so many different projects and so many things to prepare for that actually your health and wellbeing can be put on the back burner [S1004, UG3].
It’s very important. Like I said before, my body is my tool for playing the trombone, so it’s a high priority. I’ve got to look after it. I guess looking after yourself is an important thing across conservatoires [S1008, UG3].
If you’re a healthy, fit and happy musician, a happy person, then that’s going to reflect in you as a musician, and the two go very strong together and they can’t be separate things [S1004, UG3].

Additionally, a smaller number of students (*n* = 4, 20%) described the importance of knowing one’s own body:

I realized that all I had to do was listen to my body perhaps, and see if it was painful [S1004, UG3].
While playing I spend quite a lot of time kind of focused on posture and…feeling well with my instrument [S1017, UG2].

The vast majority of the students, then, report that day-to-day health and wellbeing is important to them as musicians.

Additionally, many of the students described striving to *maintain a healthy lifestyle* (sub-theme 1.2, *n* = 15, 75%). This included incorporating exercise (*n* = 11, 55%) and healthy eating habits (*n* = 7, 35%) into daily life:

Well I mean I cycle to and from College everyday. If I can’t…go for a run or go to the gym or something then at least I’m getting some form of sort of exercise. I go to the gym regularly, I have got a half-marathon on Monday, my first one. Yeah…having that as a challenge for me outside of College has been quite a big thing for me. It’s helped me massively actually [S1004, UG3].
I ran quite a lot before but I did a marathon last year. I think that had a big impact on my flute playing, breathing and then it made me think a lot about what I was eating and trying to be healthy so I could sustain all my running and also all the practice I needed to do. Whether I really think about the food that I eat so I have enough energy to last through the day and not just have spikes [S1023, UG3].

Furthermore, students demonstrated an awareness of the need to look after their health (*n* = 5, 25%) as well as the importance of regular and adequate sleep (*n* = 3, 15%).

The students also documented specific *helpful practice and learning strategies* (sub-theme 1.3, *n* = 15, 75%) that they used to support their health and wellbeing, including aiming for a broad portfolio of musical activities (*n* = 7, 35%):

I think [the conservatoire] needs to make people aware of maybe when they’re…spending too many hours locked in a practice room by themselves, and it’s not actually providing much benefit to themselves or their health really. I think trying to be aware of the different qualities that make up a good musician… . It’s not just about being technically proficient at your instrument. I think it’s about many other qualities and life experiences that go into being a good musician [S1004, UG3].
Another key moment was during my undergrad, especially with the end of my undergrad, I started to get bored with just performing with the focus on performing all the time, without the inclusion of anything else. With different modules I took here, I started to realize I could combine the more academic aspects of music with performance. I suppose that’s what I ended up doing in the end. Just realizing it didn’t have to be all one or all the other, which it felt like at some point, so it didn’t have to be that way [S1007, Doctoral student].

Additionally, students emphasized the central role of their instrumental teacher in providing day-to-day advice and support (*n* = 7, 35%):

He [my clarinet teacher] was just fantastic. A lot of my lessons in my 3rd year were me just saying, just talking to him, and he really went the extra mile. He fought for me in terms of “she can’t do this, she’s under too much pressure, she can’t do that.” Also supported me, he came and saw me in the shows, which no one else had ever done, and he said “this is your thing, I can see it’s right for you.” And that was great to have an ally in that. I’d say, yes, he was my main source of support [S1016, Graduate].
My principle study teacher, he’s quite frankly a genius and has sorted out most of my problems so far [S1008, UG3].

Further, a group of students (*n* = 5, 25%) described particular practice strategies – sometimes recommended by their teacher – that they felt supported their health and wellbeing:

I had a good talk with someone once, a teacher, where he’d gone and changed my practice technique completely… . He got me to basically, instead of feeling like you have to be this master – “you’re at conservatoire, you must be doing 4 h straight, otherwise you’re not doing your job.” That’s nonsense. That’s a very old-school way of thinking. He was like, why don’t you cut it down, do six blocks of half an hour in a day… . He was like, “you’re hindering yourself, just wearing down the fuel in your brain.” Those were the words he used. I did that, and that made a difference, feeling more positive about what I was doing. I liked it because you achieved something, almost like working your way through a checklist. You can tick them off – did that, did that, good. You’ve used that time well [S1003, UG3].

Similarly, individual students highlighted the importance of using mental practice or mental skills (*n* = 3, 15%), not practicing too much (*n* = 1, 5%), and readjusting expectations on arrival at a conservatoire (*n* = 1, 5%).

Finally, just over half of the students (*n* = 11, 55%) described ways in which they attempt to *look after their psychological health and wellbeing* (sub-theme 1.4). This sub-theme included many individual coping strategies, although there are clusters around using mental skills to achieve balance between “work and play” (*n* = 4, 20%) as well as the use of mindfulness techniques (*n* = 4, 20%):

It’s obviously important to have a balance in life. That’s something that my teacher talks about. Like I said, I’m planning my day, to have a schedule for each day, then kind of each week I plan the next week out. I plan my breaks, and I plan my having fun, and that way, when I’ve done my work, I can go and enjoy myself [S1008, UG3].
You know mindfulness meditation? I really do think that changed my life because I did it quite often but not every day. Once every few weeks or so, and then did a class every week before Christmas, but then I started taking it more seriously and actually doing it more, and after Christmas it’s completely changed the way I think. I’d say I was in a very different place than I was at this time last year [S1021, UG2].

Other students reported using relaxation techniques (*n* = 2, 10%), positive self-talk and self-belief (*n* = 2, 10%), taking time to look after and care for themselves (*n* = 3, 15%), and making use of coping strategies to help manage setbacks (*n* = 2, 10%).

To summarize this theme: the majority of students reported that day-to-day health and wellbeing is important to them as musicians. Strategies reported to enable this included a range of practice and learning strategies, such as constructing a broad portfolio of musical activities, having a supportive instrumental teacher, and developing particular practice techniques. Additionally, students reported enablers connected with maintaining a healthy lifestyle as well as finding individual strategies to promote psychological wellbeing.

#### Lifestyle Barriers

The majority of the participants also identified barriers connected with their *daily lifestyles* as conservatoire students (sub-theme 1.5, *n* = 17, 85%). These included challenges maintaining a healthy and active lifestyle such as a lack of time to eat well (*n* = 5, 25%), a lack of time to exercise (*n* = 3, 15%), and alcohol intake (*n* = 3, 15%):

Forgetting to exercise and also not eating well. Although that’s just more of a general student problem. I see it in most of my friends. When you get busy, and you have orchestra and things, especially if you don’t live very close to College, and you have just half an hour, it’s easy to go and get take away and stuff or fast food, which isn’t very good for you [S1005, UG3].
Physically, it can become a very sedentary lifestyle, and that becomes a risk… . I sat down practicing, I sat down eating, or reading. I sat down in class, I sat down chilling, I was lying down in bed. There was very little walking around [S1003, UG3].
Obviously, being a student, drinking is a part of life, and as a brass player – I was very skinny when I first came to College, but I grew a tire very quickly around the middle. Maybe, now, I’m trying to get rid of it, to be honest. I think I was quite unhealthy, to be honest, for a bit [S1008, UG3].

Additionally, the participants reported specific challenges with irregular schedules (*n* = 3, 15%), financial strain (*n* = 4, 20%), and a general sense that health and wellbeing has declined since starting at the conservatoire (*n* = 3, 15%):

It feels like more than a job. You know, like a 9-to-5 job. It’s unsociable hours and that kind of thing [S1016, Graduate].
Financial [challenge] is a big one. It’s such an amount of stress. Having your weird hours of conservatoire rehearsals and practicing and everything like that and then having to go and do a job on top of that [S1006, PG2].
I’d say [my health and wellbeing] got worse since I came [to the conservatoire] [S1001, UG3].

It is notable that the participants link the majority of the lifestyle challenges that they cite to their specific context as conservatoire students, arising as a result of busy or irregular schedules, drinking cultures, or sedentary practice behaviors. Nonetheless, although the majority of students reported one or more lifestyle challenges, these were varied across the sample and no one factor emerged as particularly dominant. Other lifestyle challenges reported included a sense that musicians are not healthy (*n* = 2, 10%) as well as struggles with the transition to the conservatoire (*n* = 2, 10%) and a lack of time for oneself (*n* = 1, 5%).

Moving to the next sub-theme, participants reported challenges associated with *physical playing-related problems* (sub-theme 1.6, *n* = 14, 70%). Of these, 10 (50%) documented experience of injury either directly or via peers:

I’ve suffered for the last 4 years from RSI [Repetitive Strain Injury], on and off. Pretty much every part of my arm has been in rather excruciating agony at some point. Basically, that’s just down to not being strong enough – core, and arms, and upper body generally, to be honest probably lower body as well – to play the trombone all day every day [S1008, UG3].
The physical thing of playing an instrument correctly and making sure it doesn’t cause damage… I know a few people that have injured themselves through over-practicing and kind of incorrect practice and that’s probably the big thing, it’s not necessarily overdoing it but doing it incorrectly [S1011, UG4].

Further, misuse of the body as a consequence of playing was associated with musicians’ health by a further five participants (25%):

We all seem to end up with posture problems from holding our violins funny or sitting at the piano or whatever. I think that’s the first thing that comes to my mind [S1006, PG2].

Finally, a further two students (10%) reported catastrophizing or negative thought patterns surrounding the acquisition of an injury:

Rather than most people who wake up a bit groggy from a cold, and realize they just need a day’s rest, they’ve just got a bit of laryngitis and maybe 3 days off singing would be helpful – I catastrophize instantly in thinking that’s it again [S1013, PG2].

It seems evident here that the body plays a central role in practice and learning, with some students reporting playing-related physical problems that may also link with negative thoughts.

Sub-theme 1.7 highlights specific *daily challenges with practice and learning* (*n* = 14, 70%). Again, there was individual variety within this sub-theme, but clusters around feelings of isolation or loneliness (*n* = 5, 25%) and challenges with the student-teacher relationship (*n* = 4, 20%):

I think musicians in general, we’re very good at isolating ourselves because we’re stuck in the practice room for, however, many hours a day [S1006, PG2].
Dealing with loneliness and the sheer isolation of [being a conservatoire student] can be quite difficult sometimes [S1003, UG3].
I don’t want to sound critical, but there are some professors who probably are not as careful about this kind of thing as they could be, both from a physical point of view of how much they push their students to do, and also from an emotional point of view, the kind of pressure they put on their students, and the expectations that they have, and maybe not communicating what exactly they mean as…clearly as they could. I know I certainly wasn’t the only one of my contemporaries to have those kind of experiences. It wasn’t at all unusual for people to feel under pressure to just constantly do more, both in terms of hours, and in terms of commitment, and all the rest of it. If you’re not clear what you’re being asked to do, then it’s very frustrating [S1007, Doctoral student].

Additional reported challenges included a feeling of pressure to be a successful performer rather than a more diversely skilled musician (*n* = 3, 15%), struggles finding balance between work and rest (*n* = 2, 10%), uncomfortable facilities in which to practice or perform (*n* = 2, 10%), and struggling with academic work (*n* = 1, 5%), technique (*n* = 1, 5%), or rehearsals (*n* = 1, 5%).

Tying together this theme, the participants identified a number of day-to-day lifestyle challenges associated with being conservatoire students as well as individual-specific challenges associated with the daily routines of practice and musical learning. Furthermore, a number of students reported barriers connected with playing-related problems such as injury or body misuse.

### Support Services Enablers and Barriers

#### Support Services Enablers

Almost all of the students felt that they had identified some adequate *support sources* to optimize their health and wellbeing (sub-theme 2.1, *n* = 19, 95%). This comprised both accessing support directly but also knowing *where* to access support, including Alexander Technique (*n* = 9, 45%), instrument teachers/coaches (*n* = 9, 45%), medical professionals (*n* = 8, 40%), or conservatoire welfare teams (*n* = 8, 40%):

[Alexander Technique], definitely. That’s all about my health and wellbeing. The sort of stereotypical thought, I guess, is, “Oh it’s just lying down and breathing, isn’t it?” No, it’s not just about sort of releasing tension and being more efficient. It’s sort of general wellbeing as well [S1008, UG3].
Sure, if I have a main issue, of course I will talk to my teacher [S1019, UG1].
I’d probably go outside of the conservatoire to my GP [General Practitioner]. If it was something specific – well, I see an osteopath reasonably regularly as well [S1010, PG3].
I’d probably go to…the head of welfare. I’d go to her, even though I’ve never spoken to her, just go use her services, so to speak. I’d probably go talk to them, yeah. That much was made very clear from day one: “If you’ve got an emotional or physical problem, I’m on the fifth floor. That’s my job. I’m here if you need me” [S1003, UG3].

A smaller number also described seeking support from counselors (*n* = 4, 20%), academic staff (*n* = 4, 20%), through yoga or Pilates (*n* = 3, 15%), movement coaches (*n* = 2, 10%), or antidepressants (*n* = 1, 5%).

In addition, over three quarters of the students (*n* = 16, 80%) reported specific *conservatoire-wide provision* for health and wellbeing (sub-theme 2.2). For the majority (*n* = 14, 70%), this emerged as a perception that the conservatoire cared for, and provided for, good health and wellbeing:

The student union does a lot to keep students happy and try to keep students healthy. We have lots of events, sponsored swims and things like that, running clubs and netball clubs… . I do think they try to make that part of our life… . Students encouraging students, rather than like staff encouraging students, because I think they feel that students would understand other students better [S1005, UG3].
They’re good at educating you here, there’s enough people you can talk to who’ve just dealt with it. Staff and other students, I wouldn’t say just staff [S1003, UG3].
I don’t think there’s any perception that we couldn’t go “I’m sorry, I’ve broken this, this hurts, I can’t do this in rehearsals today.” I never experienced any of that, and I don’t think it’s an exception for people to say, “no I can’t sing today I’ve got a sore throat.” Or “no I can’t do that action today or sing in rehearsal because my leg hurts.” Or even “no I can’t do that I’ve got my counselor’s appointment.” I think that was all catered for fairly well [S1006, PG2].

Indeed, a smaller number of students (*n* = 8, 40%) also reported specific conservatoire-based workshops or initiatives (such as performance coaching for performance anxiety and hearing health workshops) that they felt had supported their health and wellbeing.

Tying together this theme, the participants identified a number of ways in which they felt that their health and wellbeing was enabled by specific support mechanisms. These included accessing or knowing where to access adequate support sources such as Alexander Technique, instrumental teachers, medical professionals, and conservatoire welfare teams. Additionally, the majority of students felt that their conservatoire provided good care, understanding, and support for health and wellbeing.

#### Support Services Barriers

In contrast, however, over half of the students (*n* = 11, 55%) reported a perceived *lack of sufficient support* for their health and wellbeing (sub theme 2.3). Seven students (35%) explained a perceived lack of support provision:

I think [the health and wellbeing provision is] really important, but I don’t think it’s something that there’s enough of… . Really, there’s not a huge amount of health-related stuff here [S1008, UG3].
I was even a bit let down when I got tendonitis. I had never had that in my life before so I couldn’t understand. I actually expected someone to help me get through tendinitis except nobody knew what to do about it so I had to be my own doctor. Thankfully I got over that very quickly [S1014, Doctoral student].

Additionally, students (*n* = 7, 35%) reported a lack of awareness of support or where to seek help:

I don’t even know where [support] is or whether it exists, to be honest [S1010, PG3].
There doesn’t seem to be a lot at the moment. It’s getting better, but I think making students aware that there is support, like counseling and that kind of thing, I don’t think a lot of people know that that’s available [S1001, UG3].

Half of the participants (*n* = 10, 50%) also described low levels of health awareness among conservatoire students (sub theme 2.4). This emerged, firstly, as a sense that students do not maximize the health and wellbeing support on offer (*n* = 5, 25%):

I think [the health and wellbeing provision] is important, but I feel like a lot of students actually wouldn’t appreciate its relevance. When we did have days where they would give us practice, tutors coming in, or we’ve had nutritionists come in to give us lectures and talks. The turnout was just shockingly bad because the students would have rather been doing something else. They feel their health is such a low priority, or maybe they’re just unconscious that they do treat their health at such a low priority [S1005, UG3].
We had a [health and wellbeing] talk…right at the beginning. Everyone switches off, no one wants to be at these talks in the 1st week of moving to a new city. They want to be exploring or unpacking and meeting people [S1015, UG1].

Secondly, students reported that health and wellbeing is something discussed infrequently at the conservatoire (*n* = 2, 10%) or that people choose not to disclose (*n* = 4, 20%):

I guess [health is] not really talked about that much [S1022, UG2].
Well, I only told one person about [my injury], so I didn’t get help from anybody else who played [my instrument] [S1014, Doctoral student].
I don’t think there is enough. There’s still too much taboo around all kinds of health issues. I mean, I know people who have physical issues when they play but won’t address them because it might let them down in terms of their own playing and I think that just has to, has to stop because we’re young people and if we don’t sort things out now we will be in trouble for the rest of our lives [S1016, Graduate].

In sum, there seems to be a perceived lack of support for health and wellbeing as well as low awareness of both where to access support and of health promotion more generally.

### Environmental Enablers and Barriers

#### Environmental Enablers

Nineteen of the students reported enablers within the conservatoire environment. Three quarters (*n* = 15, 75%) reported experiences of *performance success and enjoyment* while studying at a conservatoire (sub-theme 3.1), and 10 (50%) identified performances that they linked with positive emotions:

I just had this feeling of such euphoria… . I really felt like I connected it. It felt totally in my body, and totally my voice connected to my body and the floor. It was an experience that I’d forgotten that you can have while singing… . That was such a good feeling. I loved it. That’s what makes me want to keep going [S1013, PG2].
There’s been a lot of great concerts that I’ve really enjoyed. I’ve done a lot of musical theater. Each concert has kind of stood out in my mind because they’ve all been wonderful experiences [S1022, UG2].

For 6 students (30%) a sense of personal progress, elicited through the conservatoire, was also important, as was, for 4 students (20%), the opportunity to focus on something that they love:

I think also just the journey I’ve been on in terms of developing as a conductor, because when I arrived I had lots of musicianship, lots of ideas, although some of that was a bit rusty through having had time out, but I’d never had any formal training… . To have learnt what I have and be in the place that I am now, and have grown in confidence, that’s also been really significant [S1010, PG3].
I think being able to come in here every day and do what you love doing, that’s an amazing opportunity [S1004, UG3].

Finally, over half of the students (*n* = 12, 60%) documented the importance of *relationships and networks* in enabling good health and wellbeing (sub-theme 3.2). In particular, students emphasized supportive conservatoire communities:

I think there’s a really nice sense of community in conservatoires…because people actually see so much of each other, they do all these rehearsals together, and a lot of performances, and a lot of projects, and a lot of different classes and small groups and things. It’s quite a social education in a way. People are generally quite supportive. I always had this fear of coming to a conservatoire, the corridors would be filled with people trying to poison each other, and stab each other in the back, and sort of filled with competitive spirit, but it wasn’t like that at all. Yes, from a positive side, it’s a good community atmosphere [S1007, Doctoral student].
I certainly feel like the people that you work with and become very close to here have been sort of a great support, and we are all to one another. You work together when you play together, but also outside of that, there’s more to it than that. I think the relationships you build up through that strengthens how you work together as well [S1004, UG3].

In addition to these relationships within the conservatoire, some students (*n* = 4, 20%) also described the importance of their parents and/or family in supporting their health and wellbeing.

In sum, the participants reported positive emotions as a result of performance experiences and progression while being conservatoire students, as well as supportive relationships and networks.

#### Environmental Barriers

All of the participants reported some degree of challenge within the conservatoire environment. The most frequently reported challenge was associated with perceived *comparison and competition* (sub-theme 3.3, *n* = 15, 75%), which seems to be a feature of the conservatoire environment in a way that the majority of students report to be emotionally challenging:

Certainly from my experience there’s been a lot of competitiveness. A lot of people are beating each other down… . We had the orchestral seating auditions and somehow people find out everybody else’s mark. I’ve no idea how. It’s just how the rumor spreads; everybody finds out everyone else’s mark and they all go, “Oh, I don’t think that person should’ve got that high” to each other. It’s like beating each other down, but it’s not actually necessarily helping yourself, it’s just lowering their self-confidence. Music is such a personal thing anyway [S1021, UG2].
I don’t actually want to be generalizing and say all musicians are highly strung and stuff, but I think it’s any performance discipline where you’re constantly putting yourself on public show, and the audience is trying to measure up to these high expectations. It’s quite stressful. That stress can cause problems for people [S1007, Doctoral student].
You’re always being listened to and when practice is quite a critical process anyway, self-critical process, I think having that constantly at the back of your mind can actually end up being quite a negative thing and you can end up becoming too self-critical of yourself as a person because it becomes very difficult to separate yourself from you as a musician and you as a human being [S1004, UG3].

Additionally, the students also described challenges with feelings of *pressure and stress* (sub-theme 3.4, *n* = 14, 70%):

The pressure, especially because it’s such a recognizable name, such a big, worldwide-known name. At the end of the day, you’re leaving here, and you’re representing their name… . You have to earn your place on the course, so then you have the pressure of maintaining and staying on it…you have to match that requirement for the next 4 years. I think that’s partly it, but also there’s a lot of work that they give you. They’re not just going to give you a degree, are they? There’s a lot of pressure [S1022, UG2].
The joy gets taken out of it because it is this pressure of “I’ve got to be able to succeed in this, otherwise my chances of being able to do this every day are sort of slightly ruined”… . It’s extremely high pressure work, and I think trying sometimes you lose sight of why you’ve chosen music and what you enjoyed about it because the pressure takes that enjoyment out of it [S1004, UG3].
Basically, in College I think people get the idea because College is so stressful, then they think when they leave College if I’m not so stressed and I’m working all the time, then I’m not doing enough. Then they kind of get used to the stress then they learn to cope with the stress, and sometimes that can be really bad [S1009, UG2].

With links to the previous sub-theme on comparison and competition, this sub-theme reveals the students’ perceptions of their conservatoire environment as intense and, at times, stressful.

Just over half (*n* = 11, 55%) of the students reported direct or indirect experiences of *psychological distress* within the conservatoire environment (sub-theme 3.5). Six students (30%) directly described an experience of mental illness:

When I went there I loved performing and just couldn’t get enough of it and then about a year and a half of being there I never wanted to step on a stage again. It was the performance class element of getting the feedback straight away. I didn’t like that at all. I actually became very depressed as a result of that [S1016, Graduate].
When I first arrived here in 1st year I was really motivated and I really absolutely loved to be in College and practice a lot, and I was in probably from half past eight in the morning until ten o’clock at night every day of the week… . I became a bit obsessive and my mental health just started to deteriorate because I have a condition, I have arthritis, well, gout. I have gout in my hands and my feet, which because of my stress levels increased due to the workload… . Then that kind of all led to a downward spiral, in a sense, until first term when I had really quite serious mental problems [S1009, UG2].

Additionally, there was also a sense (*n* = 6, 30%) that mental health can be overlooked within conservatoires:

I think those kind of movement habits and physical manifestations of health issues are much easier to stop than the mental side… . I wonder whether that side gets picked up on quite so much…because it’s not as obvious in your day to day life of the conservatoire which is playing or singing or whatever. I wonder if that gets passed over a bit more. Not that there isn’t support there but that it’s much more reliant on you realizing that you have a problem and going and seeking it out rather than someone else going “Oh your left shoulder looks a bit funny” [S1006, PG2].

Here, then, we see that mental health is a direct challenge for some students and is also perceived by others are deprioritized in relation to physical health.

For over half of the participants (*n* = 11, 55%), *challenges with performance feedback* (sub-theme 3.6) also appear to feed into constructing a challenging conservatoire environment:

The other thing is probably something that I’ve got over with time, but just particularly in that first term and that 1st year, just feeling out of my depth and a lot of anxiety about taking rehearsals, even about attending lessons sometimes, just because I just felt I wasn’t up to the task. Obviously, with feedback that you get at an early stage, or even when you swap teachers, there’s just naturally a lot of things that need to be corrected or changed, and it can feel overwhelmingly negative, however well it’s meant. I’ve gradually gotten through that. I wouldn’t have improved in the way that I have had that not been there, but I did find that very difficult, particularly the first term of that 1st year [S1010, PG3].
Ninety-nine percentage of the time… I mean I don’t do academics here so maybe I’ve got a slightly different viewpoint, but most of what you do here, you spend most of your time getting criticized for… . You don’t really improve on an instrument without being criticized, that’s the way that they teach it. Which doesn’t seem particularly helpful for your wellbeing [S1021, UG2].

Evident here is the sense that performance is “under the spotlight” at the conservatoire, whether in performance classes, rehearsals, or lessons. Indeed, students conveyed a range of individual challenges that arose as a result of specific performance situations, including feelings of anxiety about going on stage (*n* = 2, 10%) and a sense that the love of performing can be lost in an institutional setting (*n* = 2, 10%).

Finally, half of the students expressed challenges linked with *workload* within the conservatoire (sub-theme 3.7, *n* = 10, 50%). These challenges clustered onto a feeling of being overworked (*n* = 7, 35%) and the amount of practice perceived to be required (*n* = 5, 25%):

Those 6 h rehearsals in orchestra are really tough, really tough. They really can take a lot of out of you, and they can leave you quite deflated because usually they’re in the evening, or in the afternoon/evening, so they’ll start at like 2pm and finish at 9pm, and if you want to practice at all you’ll have to practice in the morning. Say you’re in orchestra and you do 4 h in the morning, and then have 6 h of rehearsal, then you’ll be practicing 10 h that day. After 10 h playing your body hurts a lot and you don’t have any time for yourself really, and that can be very detrimental [S1009, UG2].

To summarize this theme: the participants identified a number of challenges connected with the environment in which they are studying. These centered on psychologically challenging experiences of comparison and competition, as well as perceived pressure and stress. Additionally, feedback associated with specific performance experiences was reported to lead to a loss of confidence and enjoyment in performing. There was also a sense that mental health can be overlooked in favor of physical health and that workload is an additional challenge of the conservatoire environment.

## Discussion and Conclusion

**FIGURE 1:**
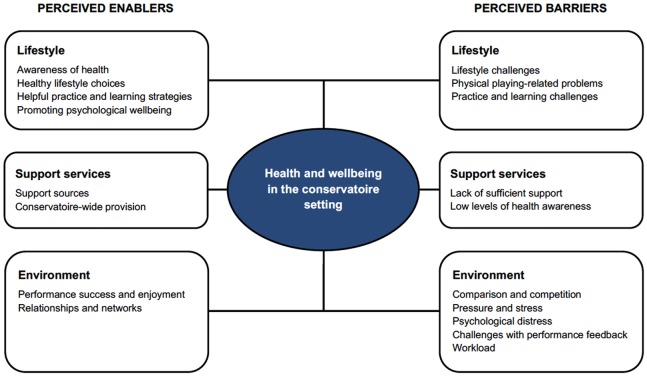
Summary of perceived enablers and barriers to optimal health among music students in the conservatoire setting.

This article investigated the following research question: How do music students in the United Kingdom perceive the enablers and barriers that they experience in relation to their health and wellbeing while studying at a conservatoire? Three sets of key enablers and barriers emerged from thematic qualitative analysis, as summarized in **Figure 1**: (i) *lifestyle enablers* included value placed on the importance of optimal health and wellbeing for musicians and daily practices to enable this; *lifestyle barriers* included struggling to maintain healthy lifestyles within the context of musical practice and learning; (ii) *support enablers* included accessible support sources within and beyond the conservatoire; *support barriers* included a perceived lack of availability or awareness of appropriate support; (iii) *environmental enablers* included enjoyable experiences of performance as well as strong relationships and communities; *environmental barriers* included experiences of comparison or competition, pressure and stress, challenges with performance feedback, psychological distress, and perceived overwork. As such, this study is the first to explore qualitatively the enablers and barriers identified by students within the United Kingdom conservatoire music setting.

These findings indicate that health promotion within conservatoires does indeed need to be understood *across* individual lifestyle, support service, and environmental conditions. While it is useful to examine individual strategies for promoting health – such as health-promoting behaviors (Kreutz et al., 2008, 2009) or performance psychology skills interventions to reduce MPA (Osborne et al., 2014) – the findings support the assertion that enquiry needs to be extended to consider the more structural components of conservatoire life, including provision available, organizational culture, and health awareness levels among students and staff. These more structural components need to be understood as complex and potentially contradictory. The students in this study experienced – within their individual programs of conservatoire study – elements of both healthy and less healthy lifestyles, adequate and inadequate support, and environmental factors that they perceived as both hindering and enabling optimal health and wellbeing. It seems reasonable to suggest, then, that perceived barriers and enablers are susceptible to change, requiring further research to engage with health determinants and outcomes as complex and multi-layered.

The balance of different health determinants across barriers and enablers is also of note. The largest number of codes concerning barriers emerged in the environmental category, relating to aspects of the conservatoire environment that were perceived as problematic for optimal wellbeing. Indeed, many of these corroborate previous research, identifying cultures of comparison or competition (Kingsbury, 2001; Davies, 2004; Juuti and Littleton, 2010) as well as challenges with psychological wellbeing connected with being a music student (Storrie et al., 2010; Hildebrandt et al., 2012). Interestingly, however, the largest number of codes concerning enablers emerged in the lifestyle category, focusing more on the individual strategies that the musicians reported using to support their own health and wellbeing. While there was evidence of environmental-level ways in which students felt their health and wellbeing was enabled – including performance opportunities that supported positive emotions and a sense of achievement (Ascenso et al., 2017) – it seems that the students more typically refer to their environment as challenging rather than enabling wellbeing. It may be, then, that the raft of individual strategies reported as lifestyle enablers arise in response to the challenging aspects of the environment, and that more attention is needed at the environmental level in order to enact sustainable health promotion change within conservatoires.

Although it was not the intention of this study to examine differences between potential participant groupings based upon characteristics such as age, sex, or musical genre, the variability found between specific factors being viewed as either a barrier or enabler of health can possibly be accounted for by other institutional and personal variables. Examining health and wellbeing provision at United Kingdom music conservatoires, Atkins (2013) noted wide variability in terms of health promoting activities across institutions as well as understanding from staff of how current health and wellbeing practices fit into larger conservatoire-wide strategy and policy. Despite the availability of a breadth of health and wellbeing support services across conservatoires, Atkins (2009) also found that, of 46 United Kingdom conservatoire students surveyed, only 43% recalled that they had received health and wellbeing education as part of their training. This points not only to the great variability between institutions in terms of how music students’ health and wellbeing is supported but also raises the likelihood that variability in awareness by the participants in the present study of available health and wellbeing enablers may have impacted upon the findings. Furthermore, even when students are aware of the availability of health and wellbeing support services, their level of engagement with such services and health information tends to be limited as reported elsewhere (Kreutz et al., 2009; Panebianco-Warrens et al., 2015). Thus, it would be relevant to investigate further the levels of health literacy among music students and how this impacts upon their perceptions and attitudes toward health and wellbeing (Nutbeam, 2000). An individual’s psychological resilience has also been found to impact upon their ability to protect themselves from stressors and influence whether a challenge is viewed as a barrier or enabler (Sarkar and Fletcher, 2014). Taken together with the present findings, this has two main implications for further research and educational development: (1) it is apparent that across conservatoires there remains a need for greater development and awareness raising of health promotion programs (enablers); and (2) more research is needed to understand better the individual characteristics and variables that impact upon musicians’ interpretation of barriers and enablers to health and their ability to make effective use of available health support resources.

There are limitations of the study that must be considered when interpreting the findings. First, the sample is biased toward women (*n* = 15 of 20), and it may be that the inclusion of more men would have changed the nature of the health and wellbeing experiences and, thus, overarching themes. Second, the sample did not include an even distribution of or representation from all instrumental and voice groups. The majority of participants identified either wind and brass (*n* = 8, 40%) or strings (*n* = 6, 30%) as their main instrument, whereas no participants identified keyboard as their main instrument. It is possible that health and wellbeing experiences may be affected by unique demands associated with different performance specialisms, which may have influenced the overarching themes. Third, the participants self-selected to take part. It is possible, therefore, that they had particularly strong experiences, either positive or negative, of health and wellbeing that they wanted to share, and that these unduly influenced the key findings. Nevertheless, the analysis procedure ensured that sub-themes were only considered as such when they were cited by at least half of the participants, thereby avoiding the possibility that dense codes on one topic from only one participant could disproportionally shape the key findings. Additionally, the thematic approach to analysis meant that individual characteristics of the students, such as their resilience, perfectionist tendencies, or general health, were not fully explored in relation to their individual experiences, and perceptions, of health and wellbeing within the conservatoire setting. Fourth, as noted within “Procedure and Methods,” the interviews varied in length. Although rich data were obtained in all interviews, it is possible that the longer interviews allowed participants to reach greater depth than the shorter interviews. Nonetheless, the interview schedule (see Supplementary Material) ensured that all participants were asked the same questions. Finally, the qualitative design, while essential in ensuring an inductive approach to this hitherto under researched topic, means that we cannot generalize the findings beyond the sample of students involved in this research.

Still, there are several implications for the field of music students’ health and wellbeing in terms of optimizing healthy lifestyles, effective health services, and healthy environments (Nutbeam, 2000). Looking first at *lifestyle*, research thus far has tended to focus on the use of the body and the impact of making music on prevalence and incidence of performance-related musculoskeletal disorders (PRMDs) (Spahn et al., 2002; Hagberg et al., 2005; Ackermann et al., 2011; Steinmetz et al., 2012). The current study suggests that music conservatoire students continue to face challenges connected with PRMDs within their daily lives and that they make use of a range of individual strategies and institutional support to prevent and cope with these. However, the range of lifestyle barriers and enablers that emerged indicates that PRMDs are but one priority for students, who are also attempting to maintain healthy eating and exercise habits and optimal psychological wellbeing within their practice and learning habits. In fact, the data imply that – as in higher education more widely (Stewart-Brown et al., 2000; Storrie et al., 2010) – psychological health is more of a concern, or at least equally of concern, than physical health. Indeed, the students recognize and value the importance of their health and wellbeing as musicians, and it appears timely to ensure that health promotion within conservatoires moves beyond a focus on the individual *body* to a focus on the individual *person* within the daily practices and routines of a conservatoire. Indeed, a recent example is the Healthy Conservatoires Network in the United Kingdom, which aims to share best practice in supporting the physical, occupational, spiritual, environmental, financial, social, intellectual, and emotional aspects of performing artists’ lives^3^. Nonetheless, the impact of such initiatives – and the extent to which students engage with them – remains to be ascertained.

Turning to *support services*, the findings demonstrate mixed perceptions from students in terms of the nature and availability of support for health and wellbeing. Many of the students show awareness of where to seek appropriate support and have benefitted from conservatoire-led provision, while simultaneously reporting that health and wellbeing support is not as developed, or as accessible, as they would like. While acknowledging that there is health and wellbeing provision in the conservatoire (e.g., lectures, workshops, services), they also imply that there can be poor engagement in some of these activities. It may be, then, that conservatoire students expect to find external answers to their problems instead of engaging autonomously in finding and using appropriate health information and support, demonstrating low levels of health responsibility (Kreutz et al., 2009). There is therefore an apparent need for continued work to embed health and wellbeing support as an integral component of conservatoire education. Linked with this is a parallel need to raise awareness of available support so that all students are fully informed of where, and how, to seek the help that they need in optimizing their health and wellbeing. In particular, the findings showed that students seek help from a range of sources, indicating that support needs to be embedded at the level of administration (in terms of advertising support and making it available), teaching faculty (in terms of providing adequate advice and support; Norton, 2016), and management (in terms of prioritizing health and wellbeing and integrating it effectively into curricula; Williamon and Thompson, 2006; Atkins, 2013).

Finally, considering the *environment*, the findings lend support to Ascenso et al.’s (2017) evidence that conservatoire students can experience positive emotions and meaning in their lives through opportunities to perform. However, some aspects of the conservatoire environment – particularly linked with challenges around performance feedback, its competitive and pressured nature, and the perceived intensive workload – emerged as challenges in terms of health and wellbeing for the students in this study. Recognizing that conservatoires’ unique learning environments and cultures can impact upon students’ approaches to and attitudes surrounding learning and performing, Papageorgi et al. (2010a,b) propose that conservatoires seek to develop positive musical learning environments that facilitate holistic development in a supportive community of learning that contribute to and reinforce students’ personal and professional identity. There is further support, then, for calls to ensure that conservatoires provide spaces for *learning* performance (Carey, 2010; Perkins, 2013b), allowing students to connect with the aspects of performance that sustain wellbeing while minimizing the negative implications of an activity that has the potential for stress and distress. Indeed, as Smilde (2009) argues, conservatoires need to provide adaptive learning environments, allowing students to mobilize their considerable initiative in terms of optimizing health and wellbeing but also working to recognize and reduce institutional constraints that may stand in their way. This means not only finding ways to recognize and address potential environmental barriers to occupational health – such as lack of funding, support, or time (Atkins, 2013) – but also scrutinizing more directly the core cultures of conservatoires and how these can be modified to best promote and optimize health and wellbeing.

## Author Contributions

RP led the conception and design of the study, data acquisition, analysis and interpretation. She drafted the article and approved the submitted version. HR contributed to the conception and design of the study, data acquisition, analysis and interpretation. She critically revised the article and approved the submitted version. LSA contributed to the conception and design of the study, data analysis and interpretation. She critically revised the article and approved the submitted version. TC contributed to the conception and design of the study, data analysis and interpretation. He critically revised the article and approved the submitted version. AW contributed to the conception and design of the study, critically revised the article and approved the submitted version.

## Conflict of Interest Statement

The authors declare that the research was conducted in the absence of any commercial or financial relationships that could be construed as a potential conflict of interest.

## Footnotes

1 www.healthyuniversities.ac.uk

2 www.healthyconservatoires.org

3 www.healthyconservatoires.org
