# Supplementary material for: Perceived Enablers and Barriers to Optimal Health among Music Students: A Qualitative Study in the Music Conservatoire Setting
Source: Front Psychol. 2017 Jun 28;8:968. doi: 10.3389/fpsyg.2017.00968 (PMC5487403; doi:10.3389/fpsyg.2017.00968)
Supplement: Supplementary file 1 [file Data_Sheet_1.pdf]

Supplementary Material

Perceived Barriers and Enablers to Optimal Health among Music Students: A Qualitative Study in the Music Conservatoire Setting

Rosie Perkins, Helen Reid, Liliana S. Araújo, Terry Clark, Aaron Williamon*

*** Correspondence:** Aaron Williamon: aaron.williamon@rcm.ac.uk

Interview Schedule

1 Introduction

- Take informed consent, reminding participant of anonymity and seeking permission to record.
- *Note for researcher:* The wording and order of the questions are flexible, and prompts/follow-up questions can be used.

2 Starter questions

- Background: Age, principal instrument, year of study.
- This interview is about your attitudes toward, and experiences of, health and wellbeing as a conservatoire student. But first, I'd like to get to know more about you and your experiences. Could you tell me about your musical development and education so far, from when you were young and until you joined the conservatoire?
  - *Prompts:* When did you start learning music? Why did you choose that instrument? What have been some of the key moments in your journey so far? How is life at the [conservatoire]?
- Now could you focus on your time at the conservatoire so far, and tell me what a typical day is like for you?
- Could you recall and tell me about one of your most positive experiences at the [conservatoire] so far?
- Could you recall and tell me about one of your least positive experiences at the [conservatoire] so far?

3 Main body

3.1 Participant's attitudes towards health and wellbeing in conservatoires

- In general, what do you feel are the most important things that a conservatoire education should provide?

- What do you think the biggest health and wellbeing issues—either positive or negative—are for conservatoire students in general?
- Could you tell me about your health and wellbeing since you started at the conservatoire? How about before you came to the conservatoire?
- How important is health and wellbeing to you as a musician? Why is this? What does this mean in terms of your day-to-day practices?
- How important do you think health and wellbeing provision is within what conservatoires offer? If important, where does it fit? And who should provide/lead it?
- Who would you approach for advice/support/help with any health and wellbeing issues which may occur?
- Could you tell me what comes into your mind when you think about musicians' health and wellbeing?

3.2 Participant's perceived *enablers* of health and wellbeing in HE

- What have you experienced since studying at a conservatoire, if anything, that you feel has supported or enhanced your health and wellbeing? What about before you came to the conservatoire?
  - *Probe:* Details, experiences, why it was supportive, who enabled it?
- What would you like to experience at a conservatoire, in an ideal world, to support and enhance your health and wellbeing? Why?

3.3 Participant's perceived *barriers* to health and wellbeing in HE

- Have you experienced or perceived any barriers to your health and wellbeing since being a conservatoire student? What about before you came to the conservatoire?
  - *Probe:* Details, experiences, why it was a barrier?
- If so, how would you propose that such barriers are removed?

4 Follow-up

- Is there anything else you would like to add to what we have discussed today?

5 Close

- Thank participant.
- Provide contact details for follow-up questions/contact.
